# A Neuro-Symbolic Bioinformatics Framework for Unlocking Chordate Physiological Dark Data and Validating Allometric Scaling

**DOI:** 10.3390/biology15090708

**Published:** 2026-04-30

**Authors:** Zhiyao Duan, Guihu Zhao, Changyun Li, Bo Liu

**Affiliations:** 1 College of Information and Intelligence, Hunan Agricultural University, Changsha 410128, China; 2 National Clinical Research Center for Geriatric Diseases, Xiangya Hospital, Central South University, Changsha 410008, China

**Keywords:** biological dark data, bioinformatics, macroecology, animal physiology, functional traits, allometric scaling, data mining, large language models

## Abstract

Animal functional trait data are essential for macroecology, but massive datasets remain locked in unstructured scientific literature. Traditional manual extraction is inefficient, and general-purpose artificial intelligence (AI) systems struggle with complex biological tables and numerical accuracy. To address this bioinformatics challenge, we propose a multimodal neuro-symbolic framework combining visual-language perception and code-based reasoning. This approach reconstructs complex document layouts and delegates biostatistical calculations, such as unit normalization and thermodynamic energy conversion, to an isolated programming environment to ensure mathematical and statistical consistency. By mining literature spanning 117 years, we constructed a high-fidelity physiological database for 1632 chordate species. Our method achieved a macro-averaged F1 score of 0.935 in extracting biophysical fields. External benchmarking against a curated mammalian trait database showed strong concordance for shared body-mass and metabolic-rate traits, while our database retained record-level provenance and physiological context. Furthermore, the extracted data reproduced classic allometric scaling relationships for basal metabolic rate and brain volume while preserving physiological adaptations, supporting the biological plausibility of the dataset. This study validates a reproducible bioinformatics pipeline that minimizes extraction artifacts and substantially reduces downstream mathematical and statistical conversion errors, while providing a scalable, complementary resource for building physiology-oriented trait databases from historical literature.

## Simple Summary

Understanding animal functional trait data is crucial for protecting biodiversity and predicting how animals will respond to climate change. However, vast amounts of valuable historical information on animal biology remain trapped in old, scanned scientific papers that lack machine readability. To address this problem, we developed a computer-assisted framework that acts like a human reader, extracting information from page layouts while using strict biological rules to ensure biophysical consistency. We used this tool to analyze over a century of scientific literature and build a physiological trait database for more than 1600 chordate species. The extracted data agreed well with established body–size relationships and with an existing curated mammalian trait database, supporting the reliability of the approach. By unlocking these hidden historical records more quickly and accurately, this framework can help scientists study animal evolution, ecosystems, and climate vulnerability.

## 1. Introduction

In the fields of macroecology and comparative physiology, functional traits of organisms encompass multiple dimensions, ranging from fundamental physiological rates to morphological characteristics, and serve as a central foundation for understanding and predicting biodiversity distribution and its responses to environmental change [1,2]. Particularly for chordates, which occupy unique evolutionary positions and exhibit highly differentiated ecological niches, their functional traits directly map the energy allocation strategies and survival adaptation potential of species in complex environments. For a long time, disciplines such as ecology, evolutionary biology, and behavioral science have heavily relied on aggregating large volumes of trait data and transforming them into structured analytical models to test ecological and evolutionary theories at large scales. For example, comparative physiological approaches have been widely applied to investigate the co-evolutionary relationships among brain size, social structure, and habitat complexity in reptiles [3,4], to quantify phenological responses of animals to global climate change [5], and to analyze the dynamic patterns of basal metabolic rates under different life-history strategies [6]. With the rapid development of large-scale ecological models and conservation biology, the use of functional trait data of chordates is becoming increasingly important in frontier research areas such as cross-species evolutionary modeling, ecosystem health assessment, and predicting wildlife vulnerability to climate change.

To date, although scientific publications have grown exponentially, the vast majority of historical physiological data remain locked within unstructured PDF documents, complex multi-page tables, and supplementary images [7,8,9]. Unstructured text has been widely recognized as a key factor causing large amounts of valuable animal physiological parameters to become effectively invisible data [10]. In fact, other scientific fields are also facing severe challenges related to data readiness [11], and across nearly all domains of science and engineering, there exists a structural dilemma of “data being available but not directly usable” [12]. This issue is particularly pronounced in biological research. Compared with fields such as high-energy physics and astronomy, biological data are inherently characterized by high heterogeneity, fragmentation, and lack of standardization, making their processing, cleaning, and integration extremely difficult [13]. This “dark data” phenomenon has forced researchers to rely on costly and inefficient manual curation for extended periods [3,7,14], significantly constraining the pace of data-driven scientific discovery. Faced with the vast reservoir of dark data embedded in biological literature, traditional purely manual management is fundamentally incapable of handling the exponentially growing volume of unstructured data, leaving domain experts trapped in tedious labor and creating an urgent need for automated extraction frameworks to break this bottleneck [15].

To address this challenge, the use of natural language processing (NLP) and large language models (LLMs) for automated literature mining has recently emerged as a highly promising bioinformatics solution [16]. However, extracting biophysical traits from historical literature is fundamentally different from ordinary text summarization. Biological data are often embedded in complex, multi-page tables or highly heterogeneous experimental descriptions, which easily confound traditional text parsing tools [17,18,19]. Moreover, constructing a rigorous functional trait database requires standardized post-processing, such as multi-source unit normalization and thermodynamic temperature conversion based on strict biophysical rules [14].

Therefore, directly applying general-purpose LLMs to scientific data mining faces three core challenges. First, the bottleneck of format parsing. Existing extraction pipelines often rely on traditional OCR combined with table-parsing heuristics or layout-aware transformers (e.g., LayoutLM). While these Document AI systems perform well on modern, standardized business documents, they often struggle with the extreme morphological degradation, severe image noise, and idiosyncratic double-column layouts characteristic of century-old scanned biological PDFs. Recently, end-to-end multimodal models have advanced document understanding by predicting Markdown formatting directly. However, they remain limited when reconstructing cross-page logical continuity and frequently suffer from structural collapse when parsing the highly dense, multi-page trait tables unique to the early physiological literature [20,21]. Gupta et al., in their large-scale study, explicitly excluded PDF documents and processed only HTML/XML, precisely because “PDFs are difficult to parse” [11]. This limitation is especially consequential in historical biology, where much of the legacy literature exists only in PDF format and text-stream parsing can easily cause row–column misalignment. Second, context fragmentation and resource inefficiency. Although recent models provide larger context windows, processing massive multi-page historical documents still often requires page-by-page processing or fixed-window chunking strategies, which introduce semantic discontinuities and computational redundancy [22]. Third, numerical randomness and lack of logical rigor. General-purpose LLMs are inherently highly non-deterministic, often producing fluctuating responses to the same prompts and lacking an intrinsic mathematical reasoning engine [23]. During multi-step numerical transformations, they are prone to “computational hallucinations.” These issues prevent LLMs from directly constructing high-confidence scientific databases.

Importantly, these methodological challenges are not unique to bioinformatics literature mining. Closely related issues have been extensively studied in radiomics and medical imaging, where quantitative pipelines must transform heterogeneous, noise-prone source data into standardized features suitable for downstream inference. In that domain, robustness depends not only on feature extraction itself, but on the full chain of preprocessing, feature definition, normalization, validation, and reproducibility control. Recent radiomics reviews, including Radiomics in Medical Imaging: Methods, Applications, and Challenges, have emphasized that feature instability, limited reproducibility, validation bias, and inadequate standardization can substantially undermine scientific generalizability even when nominal predictive performance appears strong. Likewise, our objective is not simply to parse historical PDFs, but to construct a reproducible quantitative extraction pipeline in which structured trait recovery, deterministic unit normalization, and independent validation are jointly required for trustworthy database construction. From this perspective, the present study can be situated within a broader class of established quantitative data extraction frameworks that prioritize standardization, auditability, and reproducibility across the entire analytical workflow [24,25,26].

To overcome these critical bioinformatics bottlenecks, this study proposes a multimodal neuro-symbolic framework that integrates vision-language perception with rigorous biostatistical reasoning. Rather than relying on error-prone text streams alone, the framework is built on two coordinated components. First, we developed a high-throughput, vision-first parsing pipeline that reconstructs document understanding as a computer vision task, thereby overcoming formatting-related parsing bottlenecks while preserving the topological logic of complex biological tables and fragmented ecological layouts. Second, building on the mature paradigm of decoupling semantic extraction from computation in document artificial intelligence, we implemented a deterministic biostatistical sandbox to ensure physical consistency during data processing. By delegating empirical data filtering, unit normalization, and thermodynamic energy conversion to an immutable Python-based logic engine, the framework substantially mitigates numerical hallucinations in generative models.

Based on this framework, we conducted automated fine-grained mining of historical literature spanning 117 years and constructed a high-fidelity physiological trait database covering 1632 chordate species. To evaluate the quality and utility of the resulting database, we combined multiple lines of assessment, including manual validation of extraction accuracy, external benchmarking against an established curated mammalian trait database (PanTHERIA) [27], and pattern-level biological plausibility checks based on classical allometric relationships. Through this design, we assess whether the framework can recover standardized, source-traceable, and biologically interpretable physiological records from heterogeneous historical literature. This study is therefore intended to establish a reproducible and scalable bioinformatics approach for unlocking biological dark data and complementing existing trait databases for downstream research in comparative physiology, macroecology, and global change ecology.

## 2. Materials and Methods

This section outlines our methodology for transforming unstructured PDFs into high-precision structured data through end-to-end automation and applies this approach to a fine-grained literature mining experiment on chordate physiological traits (*N* = 1632 species). We describe how each component addresses the extraction of original empirical data and the mitigation of computational numerical hallucinations, with particular attention to high-fidelity database construction. To improve reproducibility, we also report the main implementation settings, model roles, deterministic post-processing rules, and validation procedures used in the pipeline. The overall workflow integrates the large language models GLM4.6V and GLM4.7, which were accessed through the Zhipu AI/BigModel platform (Zhipu AI, Beijing, China; accessed on 19 April 2026; model names used as version identifiers), a vision-first parallel perception algorithm, and a code-enhanced deterministic verification mechanism, thereby balancing performance and computational requirements while maintaining scientific integrity through a structured validation protocol.

### 2.1. Data Corpus Construction

We constructed a curated corpus comprising core physiological journal articles published over the past 117 years (1908–2025). These articles, primarily indexed in the Crossref database and Google Scholar, were selected from authoritative journals, including Physiological Zoology, Comparative Biochemistry and Physiology, and Journal of Comparative Physiology. This dataset meticulously records key physiological parameters for 1632 chordate species, encompassing data on metabolic rates, body masses, and brain volumes.

### 2.2. Vision-First High-Throughput Literature Parsing Pipeline

**Figure 1:**
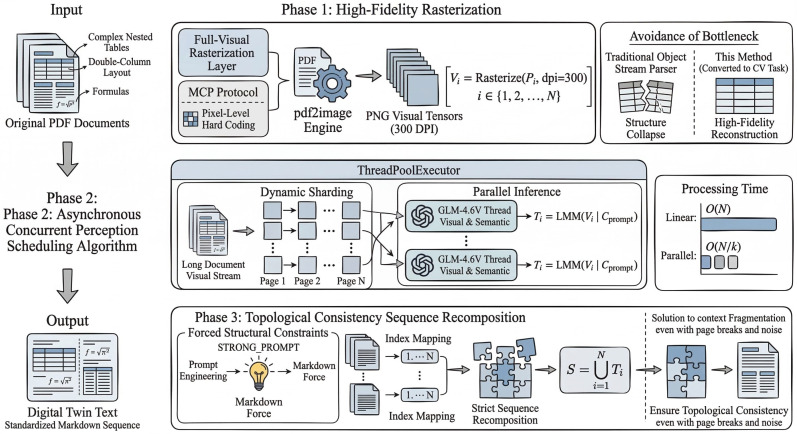
Architecture diagram of the vision-first multimodal perception system.

To effectively overcome the bottlenecks of format parsing and the limitations of context fragmentation in traditional document processing, and to achieve high-fidelity extraction of original empirical data, this study proposes a vision-first parallel full-perception algorithm. This algorithm reconstructs document parsing as a parallelized computer vision task. While significantly reducing the processing latency of long documents, it achieves high-fidelity restoration of complex nested structures and cross-page logic. The workflow of the method is illustrated in Figure 1.

#### 2.2.1. High-Fidelity Restoration of Biological Literature

Traditional parsers often lead to structural collapse when extracting complex nested tables, double-column layouts, and formulas commonly found in biological literature. To prevent the loss of critical morphological and physiological data, this algorithm constructs a parsing pipeline based on the MCP (Model Context Protocol) to overcome the bottlenecks of PDF parsing. It leverages the pdf2image engine to forcibly map PDF documents into 300 DPI lossless PNG visual tensors. This process ensures high-fidelity restoration of table boundaries and mixed text-image layouts.

#### 2.2.2. High-Throughput Parallel Perception Strategy

Multimodal large models (LMMs) exhibit significant latency and context window limitations when processing long documents, and linear processing leads to extremely low efficiency. To address this, we designed a high-throughput parallel inference strategy based on ThreadPoolExecutor, which is provided by the concurrent.futures module in the Python standard library (Python 3.8+).

1. Dynamic sharding: The algorithm discretizes long documents into independent visual units, enabling dynamic segmentation.
2. Parallel inference: Leveraging the concurrency capabilities of the MCP server, multiple LMM (GLM-4.6V) inference threads are instantiated simultaneously to perform parallel visual encoding and semantic extraction on different pages of the document. This approach compresses the original linear O(N) processing time to O(N/k), where k is the number of concurrent threads, effectively resolving the computational bottleneck of deploying complex models on large-scale ecological datasets.

In implementation, each PDF page was rendered independently at 300 DPI and processed as a single visual unit. To reduce variance in model outputs, page-level extraction was constrained to a standardized structured template rather than free-form prose. All page outputs were collected as intermediate structured text blocks before reconstruction. When malformed or incomplete outputs were detected, the corresponding page was reprocessed under the same schema-constrained settings and logged for later inspection if the problem persisted. The degree of concurrency was controlled at the workflow level rather than left unconstrained, and the same structured extraction template was applied uniformly across all pages to reduce run-to-run variability.

#### 2.2.3. Topological Consistency Sequence Reconstruction

Traditional RAG (Retrieval-Augmented Generation) approaches often break the logical continuity of cross-page tables and paragraphs during the chunking process, leading to context fragmentation. To maximize the integrity of the extracted raw data, this tool introduces a sequence-consistent reconstruction mechanism. Through structured prompt constraints, the system enforces the model to output data in a standardized format, followed by strict sequence reconstruction based on page topological order. The result is a text output that preserves complete semantics and layout structure. Even when dealing with image noise and table distortions in legacy PDF documents, the method achieves high-fidelity reconstruction of tabular data, thereby maximizing the completeness of original empirical data extraction.

This algorithm packages all these processes into a streaming execution framework via the MCP protocol, enabling intelligent agents to automatically identify and invoke it. By leveraging visual tensor mapping and parallel computation, the algorithm achieves efficient processing while ensuring topological consistency. Its core formalized execution logic is as follows:

First, in the high-dimensional visual mapping stage, the *i*-th page $p_{i}$ of a long document is mapped to a high-resolution visual tensor $V_{i}$:

(1) $$V_{i}=\mathrm{Rasterize}\left(P_{i},\;\mathrm{dpi}=300\right),\;\;i\in \{1,2,...,N\}$$

Second, in the constrained concurrent perception stage, the system launches k concurrent threads and uses the multimodal large model (LLM) to extract textual representations $T_{i}$ in parallel. This extraction process is strictly constrained by the strongly structured prompt $C_{\mathrm{prompt}}$:

(2) $$T_{i}=\mathrm{LMM}(V_{i}|C_{\mathrm{prompt}})$$

Finally, in the topological sequence aggregation stage, the system performs sequence reconstruction of the results according to the topological order of the original page numbers, producing a structured aggregate *S* with complete contextual semantics:

(3) $$S=\overset{N}{\underset{i=1}{⋃}}T_{i}$$

In the reconstruction stage, page outputs were merged strictly according to the original page index and the preserved intra-page reading order. Cross-page tabular continuity was resolved conservatively: only adjacent page outputs showing clear structural continuation were merged, whereas ambiguous boundaries were retained as separate segments to avoid artificial concatenation. This design prioritizes structural fidelity over aggressive recovery and makes the reconstruction procedure more reproducible across documents with heterogeneous layouts.

### 2.3. Code-Enhanced Deterministic Verification Mechanism

**Figure 2:**
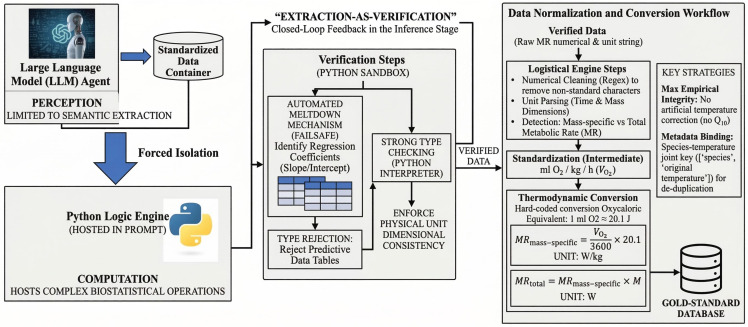
Framework diagram of the code-enhanced deterministic verification mechanism.

To overcome the challenges of probabilistic hallucinations and logical non-determinism of large language models in numerical processing, this study introduces an automated data quality control paradigm. All complex biostatistical computations are delegated to a deterministic external host environment, thereby strictly constraining the model’s natural language generation. The workflow of the method is illustrated in Figure 2.

#### 2.3.1. Decoupling of Perception and Computation

This framework designs a strict “data–logic isolation layer.” In the data extraction workflow, the agent is strictly restricted to functioning only as a semantic extraction “perceiver,” responsible for populating standardized data containers, while complex biostatistical computations (such as unit normalization and thermodynamic energy conversion) are entirely delegated to an immutable Python 3.8-based logic engine embedded within the prompt.

#### 2.3.2. Automated Quality Control and Filtering of Non-Empirical Data

This mechanism introduces an “extraction-as-validation” closed-loop feedback during the inference stage. The system incorporates automated quality control and rigorous filtering mechanisms tailored for the biological literature: when the agent detects regression coefficients within table structures (such as slopes and intercepts in allometric models), it immediately triggers type rejection, automatically excluding these non-original, model-derived data tables. In addition, the system enforces strict physical unit consistency through rigorous type checking. This approach transforms non-deterministic natural language generation into deterministic code execution, providing an engineering-level mechanism for significantly reducing numerical hallucinations.

#### 2.3.3. Heterogeneous Data Normalization and Thermodynamic Energy Conversion Model

Chordates encompass a vast body size range, spanning from gram-scale miniature amphibians to hundred-kilogram-scale large reptiles. Over a century of historical literature, the recording units for their physiological parameters exhibit extreme fragmentation (for example, early literature frequently used $\mathrm{cc}\;O_{2}$ instead of $mL\;O_{2}$, and absolute metabolic rates are widely intermixed with mass-specific metabolic rates). To endow the system with the generalization capability to process such full-lineage, cross-era literature, the system’s sandbox logic engine incorporates built-in conditional routing and strict thermodynamic conversion operators.

When processing metabolic rate data, the system first cleans non-standard characters in the data (such as confidence intervals or parenthetical annotations) through regular expressions and then parses the original unit strings extracted by the large model. The logic engine automatically determines whether the value represents a mass-specific metabolic rate or a whole-body metabolic rate by detecting the mass and time dimensions within the string and then aligns all oxygen consumption data to the standardized intermediate metric of $mL\;O_{2}\;kg^{-1}\;h^{-1}$. Subsequently, based on the standard oxygen caloric equivalent of 20.1 J mL^−1^ O_2_, a commonly used conversion factor in metabolic-rate studies, the system converts oxygen consumption into absolute energy power (Watts) through hard-coded thermodynamic transformation.

The underlying thermodynamic conversion formulas for mass-specific metabolic rate $\left(MR_{\mathrm{mass}\text{-}\mathrm{specific}}\right)$ and whole-body total metabolic rate $\left(MR_{\mathrm{total}}\right)$ executed within the sandbox are as follows:

(4) $$MR_{\mathrm{mass}\text{-}\mathrm{specific}}=\frac{V_{O_{2}}}{3600}\times 20.1$$

(5) $$MR_{\mathrm{total}}=MR_{\mathrm{mass}\text{-}\mathrm{specific}}\times M$$

Here, $V_{O_{2}}$ represents the standardized oxygen consumption rate (unit: $mL\;O_{2}\;kg^{-1}\;h^{-1})$, 3600 is the time conversion coefficient from hours to seconds, and M represents the normalized body mass (unit: kg). The final output unit of $MR_{\mathrm{mass}\text{-}\mathrm{specific}}$ is W/kg, while the unit of $MR_{\mathrm{total}}$ is W.

It is worth noting that, in order to preserve the empirical purity of historical data to the greatest extent possible, the system does not apply empirical formulas to perform artificial temperature-based corrections. Instead, the large model precisely extracts the original experimental temperatures specified in the literature, and during structured output, deduplication and strict metadata binding are performed using the composite primary key [‘species’,‘originaltemperature’]. This strategy not only accommodates the complex experimental designs present across most chordate taxa but also effectively controls/minimizes artificial conversion errors and dimensional inconsistencies in cross-species comparisons, ensuring that the final high-fidelity database remains closely aligned with the original measured values.

To improve reproducibility, the main deterministic operations of the sandbox were explicitly fixed rather than inferred at runtime. These operations included rejection of regression-derived parameters, regular-expression cleaning of non-standard numeric annotations, rule-based interpretation of metabolic-rate unit strings, thermodynamic conversion of oxygen-consumption values into standardized energy units, brain-size normalization across mass and volume units, and record deduplication based on species and original temperature.

### 2.4. Extraction Accuracy and Consistency Evaluation Strategy

This study adopts a hybrid validation strategy combining “sandbox strict verification” with “manual blind sampling review.” First, all extracted data must pass the physical consistency checks and type-based circuit-breaking in the Python sandbox (see Section 2.3.2). Subsequently, a validation subset was randomly sampled from the final system output dataset, comprising 100 independent publications. To ensure objectivity, two independent researchers, without access to the model outputs, performed data provenance tracing and manual extraction using a predefined annotation guideline. Disagreements after independent annotation were resolved through consensus review. To comprehensively evaluate extraction performance and address the issue of class imbalance in historical corpora, this study establishes a clear complexity stratification for “core fields”:

1. Low complexity: Taxonomic entities (e.g., species scientific names), which typically appear frequently in the text and follow fixed formats.
2. Medium complexity: Context-linked physical and experimental traits (e.g., body mass and experimental temperature), which require associating numerical values with units across local contextual spans.
3. High complexity: Thermodynamic parameters requiring deterministic conversion (e.g., normalized metabolic rate), which are sparse and often require cross-page association, unit interpretation, and composite thermodynamic transformation within the sandbox.

This stratification strategy clarifies the weighting of macro-averaged F1 scores when handling extreme class imbalance, ensuring that rare high-complexity features are fairly represented in the overall evaluation. Evaluation metrics focused strictly on the core fields, including precision, recall, and F1 score calculations. Furthermore, 95% confidence intervals (CIs) for all performance metrics were estimated using t-based intervals across the five independent validation samples to reflect variation in performance across repeated sampling. Conceptually, this evaluation design follows the same end-to-end logic emphasized in quantitative radiomics pipelines, in which extraction quality is assessed not as an isolated parsing task but as a function of standardized preprocessing, explicitly defined feature transformation, and independent validation. We therefore treat provenance tracing, deterministic normalization, and external concordance analysis as integral components of reproducibility rather than optional downstream checks [24,25,26].

### 2.5. Data Serialization and Structured Persistence Layer

We performed lightweight serialization using the Pandas data analysis library (version 2.0.3). The system automatically generates conflict-free, secure filenames based on the extracted metadata. Prior to persistent storage, the cleaning pipeline applies strict deduplication and merging operations on the dataset based on composite primary keys, ensuring the uniqueness of physiological records for the same species under identical test temperatures. The final structured dataset is exported in a standardized CSV format with UTF-8-SIG encoding, seamlessly supporting downstream large-scale evolutionary analyses using commonly used R language or Python statistical packages in macroecology [28,29]. The exported CSV files were designed for compatibility with downstream R- and Python-based statistical workflows; however, no specific R package was used during this serialization step. The serialization and cleaning procedures in this study were performed using Python 3.8.10 and Pandas 2.0.3. Distinct measurements were retained as separate records when they differed in experimentally relevant metadata or source context, whereas only duplicate entries sharing the same composite key and identical extracted values were merged during the cleaning stage.

### 2.6. Computational Environment and System Implementation

All local structured parsing, high-fidelity rasterization, and Python-based sandbox verification logic in this study were executed on a local computer equipped with an AMD Ryzen 7 8845H processor with integrated AMD Radeon 780M Graphics (Advanced Micro Devices, Inc., Santa Clara, CA, USA), 32 GB of RAM, and running Microsoft Windows 11 (Microsoft Corporation, Redmond, WA, USA). The core workflow was implemented in Python 3.8.10 (Python Software Foundation, Wilmington, DE, USA), with inter-module communication and tool invocation handled through the MCP (Model Context Protocol) framework. At the document preprocessing level, high-resolution visual tensor conversion relied on the system-level rendering engine Poppler (version 23.11.0; freedesktop.org; accessed on 19 April 2026) and the Python wrapper libraries pdf2image (version 1.17.0) and Pillow (version 10.4.0), while document metadata extraction was driven by PyPDF2 (version 3.0.1).

In the current implementation, GLM-4.6V and GLM-4.7 served as the model backends for visual parsing and structured reasoning, respectively. These proprietary model backends were accessed through the Zhipu AI/BigModel platform (Zhipu AI, Beijing, China; accessed on 19 April 2026), and the model names GLM-4.6V and GLM-4.7 represent the version identifiers used in this study. In practical terms, the vision-capable model was used for page-level perception and structured extraction from rendered document images, whereas the reasoning model was used for constrained text interpretation and interaction with the deterministic sandbox.

### 2.7. Reproducibility and Implementation Details

To improve technical reproducibility, we explicitly report the main implementation logic of the framework. PDF documents were rendered page by page at 300 DPI and processed as independent visual units. Structured extraction was performed under prompt-constrained settings, and all numerical standardization, empirical-data filtering, and thermodynamic conversion were executed by a deterministic Python sandbox implemented in Python 3.8.10 rather than by the language model itself. The sandbox was a custom rule-based execution environment developed for this study, not a separately released software package. Reproducibility in this framework depends primarily on preserving the same page-level rendering protocol, structured extraction template, deterministic sandbox rules, and validation procedure, rather than on replicating any single model backend verbatim. Although the current implementation relies on proprietary models, the framework is modular in design, and future work will evaluate additional open-source and commercial backends to further assess reproducibility and generalizability across models.

## 3. Results

### 3.1. Automated Construction of a Chordate Physiological Trait Database

**Figure 3:**
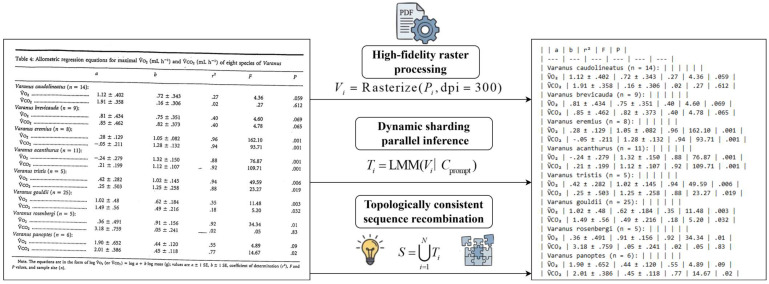
Data conversion diagram for legacy PDF documents.

We transformed unstructured PDF documents of chordate physiology into high-precision structured data, achieving an end-to-end automated process. The system begins by importing PDF documents, and the agent automatically preserves and converts the text, images, and tables within the documents into Markdown sequences, ensuring the retention of all visual elements. Figure 3 demonstrates that even when faced with image noise and table skew distortions in outdated PDFs, the system can still achieve high-fidelity reconstruction of table data. (This figure is intended solely to demonstrate the system’s capability to parse complex layouts. In the final database construction, the contents of such tables are identified and excluded by the logic engine and are not included in the final set of 2192 records).

**Figure 4:**
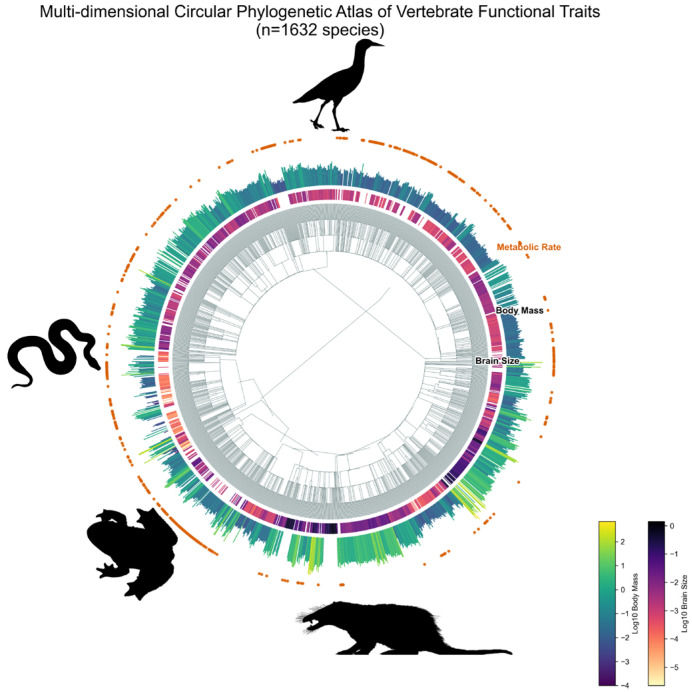
Multidimensional circular phylogenetic tree.

Using the “vision-perception and code-enhanced reasoning collaboration” multimodal intelligent agent framework proposed in this study, we successfully performed end-to-end automated fine-grained mining of the core physiology literature published over the past 117 years. The system ultimately extracted and constructed a standardized physiological trait database encompassing 1632 chordate species (see Figure 4).

In the data processing pipeline, the sandbox-level automated circuit-breaking mechanism played a critical role. During the processing of the original corpus, the system successfully identified and triggered 427 removal operations targeting predicted data from regression models or allometric growth equations, effectively preventing non-empirical data from contaminating the final database. These excluded entries were logged during processing but were not retained in the released empirical database because they represent model-derived quantities rather than direct physiological observations. All retained valid records successfully passed the Python logic engine’s strict type checks, underwent physical quantity normalization based on the oxygen caloric equivalent, and ultimately yielded 2192 high-quality species physiological trait records (see Table 1).

In terms of extraction completeness, this framework demonstrated strong metadata binding and contextual retrieval capabilities. The extraction coverage for basic taxonomic information (such as order, family, genus, and species) and reference provenance reached 100%, providing a solid phylogenetic foundation for subsequent macroecological and evolutionary analyses [30].

In the mining of core physiological and morphological traits, body mass, and brain volume, as the most fundamental biophysical parameters, were successfully extracted for 1968 and 1672 valid records, with coverage rates of 89.8% and 76.3%, respectively. Notably, metabolic rate and its corresponding experimental temperature, as deeply nested parameters often embedded within complex experimental protocol paragraphs and cross-page tables, were still successfully recorded for 516 and 405 entries after strict normalization by the thermodynamic engine. This distribution pattern accurately reflects reporting practices in historical physiological literature: compared with readily obtainable traits such as body length or brain volume, live metabolic rate measurements are more challenging to obtain [31], making the data considerably scarcer. However, leveraging the system’s underlying sandbox circuit-breaking mechanism, all 516 metabolic rate records were successfully converted into absolute energy units (Watts and W/kg), achieving dimensional consistency and physical rigor [32]. Importantly, beyond single-field coverage, the resulting database retains a substantial number of analysis-ready trait combinations, especially for records linking body mass with brain volume or metabolic rate, and for a subset of metabolic observations that also preserve experimental temperature. This combination-level structure increases the practical utility of the database for downstream comparative and physiological analyses. To further assess the external validity and downstream utility of this database, we next benchmarked its mammalian subset against an established curated trait resource.

### 3.2. Benchmarking Against an Established Curated Mammalian Trait Database

To provide an external benchmark against an established curated resource, we compared the mammalian subset of our database with PanTHERIA 1.0 (WR05 release, August 2008), a widely used species-level mammalian trait database containing body mass and metabolic rate information [27].

#### 3.2.1. External Comparison Design

PanTHERIA is particularly suitable as an external comparator because it provides consolidated mammalian trait values, including body mass and metabolic rate, thereby enabling direct comparison with the two most comparable physiological traits in our mammalian subset.

Because the released PanTHERIA table reports one consolidated value per species, whereas our database preserves multiple observation-level records for the same taxon, we first aggregated our mammalian records to the species level before comparison. Specifically, repeated measurements within each mammalian species were summarized using the median, which also aligns with the consolidation philosophy used in PanTHERIA for continuous variables. We then performed exact binomial-name matching between the two resources and quantified agreement for body mass and absolute metabolic rate using log-scale correlation and deviation metrics.

Our mammalian subset contained 1020 record-level observations representing 675 species, whereas the released PanTHERIA table contained 5416 mammalian species-level rows. Exact binomial-name matching identified 541 shared species, corresponding to 80.1% of the mammalian species in our database. Among these shared taxa, 475 species had directly comparable body-mass values and 81 species had directly comparable metabolic-rate values after harmonization to the same energy unit, with PanTHERIA providing species-level basal metabolic rate estimates for these matched comparisons. Because no synonym reconciliation was applied, the overlap reported here should be regarded as conservative. The structural differences and complementary characteristics of the two resources are summarized in Table 2.

#### 3.2.2. Trait Concordance on Shared Species

Across the matched mammalian species, our aggregated species-level values showed strong concordance with PanTHERIA for the traits that were directly comparable. For body mass, comparison across 475 shared species yielded a Pearson correlation of 0.988 and a Spearman correlation of 0.987 on the log10 scale. The median absolute log10 difference was 0.081, corresponding to a median fold difference of 1.21, and the fitted log-scale slope between the two resources was 0.972. These results indicate that the body-mass values recovered by the automated extraction and normalization pipeline were highly consistent with those in a classic manually curated mammalian database.

Agreement was even stronger for metabolic rate. After converting PanTHERIA basal metabolic rate values from $mL\;O_{2}\;kg^{-1}\;h^{-1}$ to Watts using the same oxygen caloric equivalent adopted in our pipeline, comparison across 81 shared species yielded a Pearson correlation of 0.996 and a Spearman correlation of 0.995 on the log10 scale. The median absolute log10 difference was only 0.010, corresponding to a median fold difference of 1.02, and the fitted log-scale slope was 1.009. This close agreement suggests that the deterministic standardization framework recovered species-level metabolic information that is numerically highly concordant with an established curated database.

**Figure 5:**
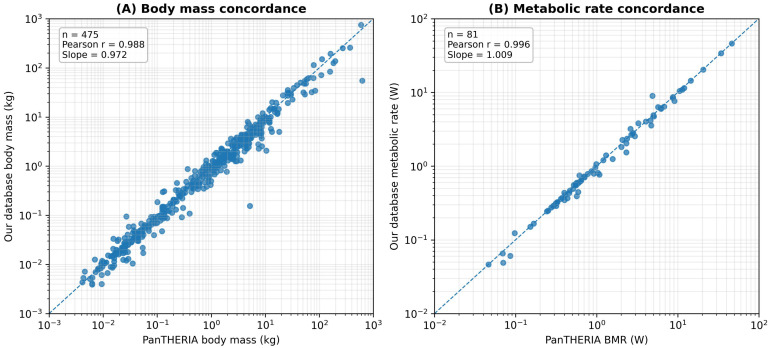
Concordance between species-level estimates from our mammalian subset and PanTHERIA. Blue dots represent shared mammalian species with directly comparable trait values between the two databases. The dashed line indicates the 1:1 reference line, where the species-level values from our database are equal to the corresponding values from PanTHERIA.

Taken together, these results show that the present extraction pipeline does not merely reproduce broad biological trends at the pattern level but can also recover trait values that closely match those of a canonical species-level mammalian resource on shared taxa. The close agreement in species-level body mass and basal metabolic rate between the two databases is visualized in Figure 5. The corresponding quantitative concordance statistics are reported in Table 3.

#### 3.2.3. Complementary Value of Record-Level Physiological Metadata

Despite this strong species-level agreement, the present database differs from PanTHERIA in an important way: it preserves record-level provenance and physiological context rather than only a single consolidated value per species. Within the mammalian subset alone, our database retained 1020 record-level observations for 675 species, including 935 standardized body-mass records, 177 standardized absolute metabolic-rate records, 839 brain-size records, and 123 metabolic observations with retained original experimental temperature.

In addition, each mammalian record in our database remained linked to its original reference, publication year, and sample size metadata, while also preserving the original measurement fields and original unit strings alongside the standardized output values. This structure differs fundamentally from a species-level summary table. Rather than collapsing all source measurements into a single released trait value, the present resource retains the measurement context needed for direct source tracing, auditability, and reinspection of historical literature.

This additional granularity is particularly valuable for physiological data. Unlike morphology-only summaries, metabolic measurements are sensitive to experimental conditions, measurement conventions, and unit heterogeneity. By preserving both standardized outputs and source-level metadata, the present database provides a more transparent bridge between legacy literature and downstream quantitative analysis.

#### 3.2.4. Implications for Downstream Use

The comparison with PanTHERIA indicates that the present database complements, rather than replaces, a classic mammalian species-level resource. PanTHERIA is suitable for broad comparative analyses requiring one standardized species value per mammal, whereas our database preserves record-level observations, original experimental temperature, and source-linked metadata. This retained structure supports several downstream uses. First, it enables measurement-aware meta-analysis rather than reliance on a single species summary value alone. Second, the preservation of original experimental temperature allows condition-aware reinspection of metabolic records derived from heterogeneous historical literature. Third, the explicit linkage between each record and its original reference supports reproducible source auditing and targeted manual verification when needed. Accordingly, the present resource adds record-level detail, source traceability, and experimental context to species-level mammalian trait resources such as PanTHERIA.

### 3.3. Biological Plausibility Check Using Classical Allometric Relationships

Following the evaluation of extraction accuracy and external concordance with an established curated mammalian trait database, we further examined whether the final dataset preserved well-known macroecological and physiological scaling patterns. In vertebrates, basal metabolic rate and brain volume are expected to scale with body mass according to classical allometric power-law relationships, expressed as $Y=aM^{b}$, which appear as linear trends on log–log axes [33]. Here, these relationships were used as a secondary plausibility check of the extracted dataset rather than as a sole validation of database quality. In the present study, these relationships were estimated using ordinary least squares (OLS) on the extracted cross-species records and were intended as descriptive plausibility checks rather than phylogenetically corrected comparative inferences.

**Figure 6:**
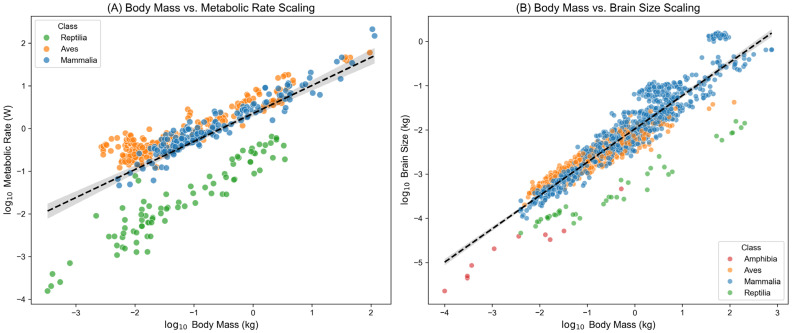
Allometric scaling relationships for metabolic rate and brain volume. Colored dots represent individual extracted records from different animal classes. The black dashed lines indicate the fitted ordinary least squares regression lines on log–log axes, and the grey shaded areas represent the 95% confidence intervals around the fitted regressions.

As shown in Figure 6A, we performed regression analysis on 516 metabolic-rate records that were automatically extracted and standardized by the sandbox into absolute units (kg and W). Across the full sample spanning Reptilia, Aves, and Mammalia, log-transformed body mass and log-transformed absolute metabolic rate showed a clear linear relationship (R^2^ = 0.515), with an overall scaling slope of 0.656. This estimate is close to the classical surface-area expectation of approximately 0.67 [34]. Within endotherms, the Mammalia subset yielded a slope of 0.671 (R^2^ = 0.910), again consistent with established biological expectations.

Similarly, analysis of 1672 valid brain-volume records (Figure 6B) showed that the cross-species brain–body scaling slope reached 0.753 (R^2^ = 0.854), which is broadly consistent with classic findings in comparative neurobiology [35,36]. Together, these results indicate that the extracted dataset preserves large-scale biological structure expected from vertebrate trait data.

Importantly, these allometric patterns should be interpreted as pattern-level plausibility checks rather than definitive evidence of database superiority, because such relationships can be robust to moderate noise or sampling bias. Accordingly, stronger support for database quality comes from the combination of high field-level extraction accuracy, deterministic unit standardization, preservation of source-level metadata, and concordance with PanTHERIA on shared mammalian traits. Because phylogenetic non-independence was not explicitly modeled, the reported slopes should not be interpreted as phylogenetically independent comparative estimates.

### 3.4. Evaluation of Extreme Value and Biodiversity Capture Capability

**Figure 7:**
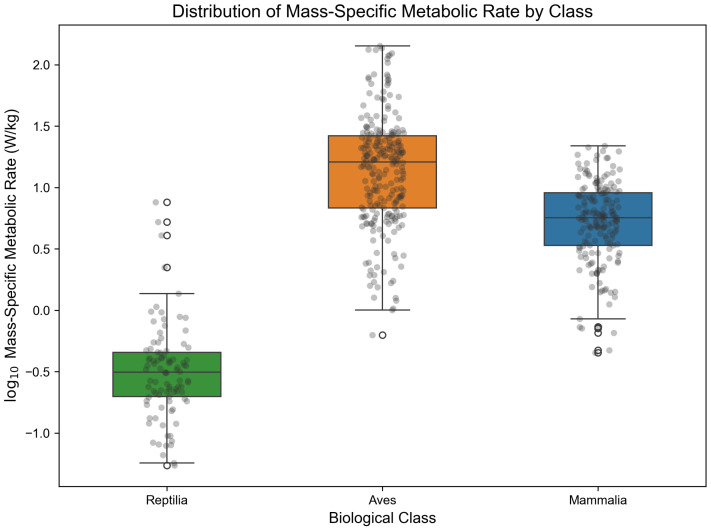
Boxplot of mass-specific metabolic rate distribution and extreme value traceability across animal classes. Colored boxes show the interquartile range and median values for each class. Grey dots represent individual extracted records plotted with jitter to show the distribution of observations, whereas white hollow dots indicate statistical outliers identified by the boxplot algorithm.

Complex unstructured historical literature often contains exceptional cases and extreme physiological values found in nature. To assess whether the code-enhanced sandbox might erroneously filter out genuine biological extreme data due to an overly strict “circuit-breaking mechanism,” this study conducted an in-depth traceability analysis of the mass-specific metabolic rate distribution in the final database (Figure 7) [37].

The analysis results show that the system successfully captured and accurately converted the natural limits of energy metabolism spanning nearly four orders of magnitude. For example, within Aves, the system precisely extracted and retained hummingbird species with extremely high metabolic costs, such as the scintillant hummingbird and the violet-crowned hummingbird, whose mass-specific metabolic rates reached as high as 142.5 W/kg and 136.9 W/kg, respectively, consistent with the extraordinary energy demands of hovering flight. In contrast, within Reptilia, the system also accurately identified the metabolic minima of ectothermic animals, such as the alligator snapping turtle and various monitor lizards, with rates as low as approximately 0.05 W/kg [38].

This strong tolerance for the long-tail distribution of physiological traits, together with high-fidelity reconstruction capability, further supports the robustness of the “vision-driven large model + deterministic Python logic engine” paradigm. The system not only effectively filters out the common “equation-intercept hallucinations” of large models, but also faithfully preserves genuine biological diversity outliers [39].

### 3.5. Engineering Efficiency and Structural Reconstruction Performance of the Parsing Pipeline

All baseline comparisons were performed on the same document subset and computational environment, using fixed settings for each pipeline to ensure comparability. To evaluate the performance of the proposed vision-first concurrent parsing pipeline on complex scientific PDFs, we compared it with two baseline categories:

1. Traditional OCR and heuristic table-parsing pipelines, represented here by pdfplumber (version 0.11.4);
2. Standard serial multimodal-model pipelines, represented here by direct GPT-4o processing through the OpenAI platform (GPT-4o, model version gpt-4o-2024-08-06; OpenAI, San Francisco, CA, USA; accessed on 19 April 2026) with standard prompting and without parallel scheduling or sequence-reconstruction constraints.

Because this section evaluates engineering efficiency and document reconstruction quality rather than binary classification performance, ROC/AUC-style metrics are not applicable and were therefore not reported.

**Figure 8:**
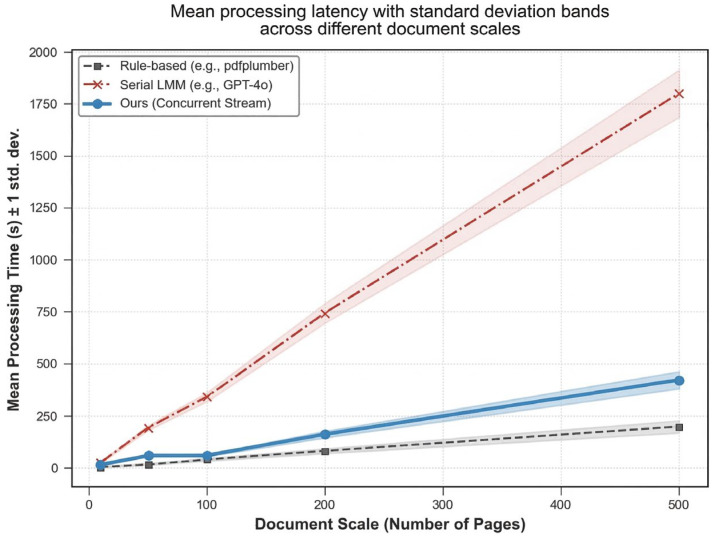
Time Cost Analysis Line Chart.

In the time cost analysis (Figure 8), the conventional pure large-model serial pipeline showed a marked increase in latency as document length increased, consistent with its reliance on long-context processing [22]. In contrast, the proposed concurrent parsing strategy maintained substantially lower processing time while preserving linear scalability across increasing page numbers. Although the framework includes computationally intensive visual rasterization and multimodal inference, its overall runtime remained close to that of lightweight traditional parsing tools, supporting its feasibility for high-throughput literature mining.

**Figure 9:**
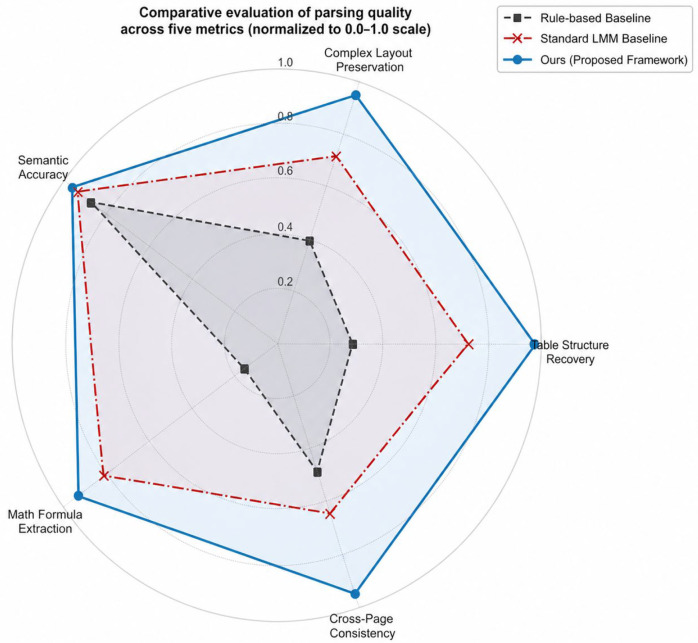
Comprehensive quantitative evaluation radar chart.

We further compared the three pipelines across five document-reconstruction dimensions: table structure restoration, layout preservation, semantic accuracy, formula parsing capability, and cross-page logical consistency (Figure 9). The proposed framework showed the most balanced overall profile across these dimensions. Traditional Python parsing tools performed less reliably on degraded or non-standardized layouts, especially for table structure restoration, whereas the vision-first strategy improved preservation of table boundaries and cell alignment [21]. Standard serial multimodal-model pipelines also showed weaker performance on long tables and cross-page passages, where chunk-based truncation can introduce semantic discontinuities [40]. By combining page-level perception with sequence-consistent reconstruction, the proposed framework preserved more of the topological structure and contextual continuity of cross-page scientific data.

### 3.6. System Evaluation and Method Comparison

The manual validation subset was independently cross-checked by two researchers following the predefined annotation guideline described in Section 2.4. Since the objective of this study is to construct a high-fidelity animal physiological database, relying entirely on large language models (LLMs) for automated cross-validation may introduce secondary hallucination biases. Therefore, this study adopts a hybrid validation strategy combining “sandbox strict verification” with “manual blind sampling review.”

First, all extracted data must pass the physical consistency checks and type-based circuit breaking in the Python sandbox (see Section 2.3.2). Subsequently, from the final dataset produced by the system, a validation subset was selected through five independent random samplings (each with *N* = 20, totaling 100 independent publications). Researchers, without access to the outputs of the large model, directly compared the original PDF literature for data provenance tracing and conducted independent manual extraction. They then manually calculated the standard information retrieval metrics—precision, recall, and F1 score—for each target feature field. The validation process employed strict semantic and dimensional matching rather than naive string comparison (for example, treating an extracted value of 5000 g as equivalent to a manually annotated value of 5 kg). Table 4 presents the aggregated performance metrics, revealing the decisive impact of introducing a deterministic code sandbox on the accuracy of scientific knowledge extraction.

Overall, the multimodal neuro-symbolic framework proposed in this study demonstrates statistically significant performance advantages across multiple independent sampling tests. The overall macro-averaged F1 score reaches 0.935 (95% CI: 0.928–0.942), representing a highly significant improvement compared to the pure large-model baseline (F1 = 0.780, 95% CI: 0.766–0.794, *p* < 0.001). A detailed analysis of specific data fields reveals the destructive impact of “calculation hallucinations” on scientific extraction. When extracting simple and well-defined discrete entities (such as species scientific names), both the pure LLM and the proposed framework perform excellently, with F1 scores of 0.948 (95% CI: 0.934–0.962) and 0.971 (95% CI: 0.962–0.980), respectively, which is consistent with the well-known advantages of modern LLMs in standard named entity recognition (NER) tasks [41].

However, when dealing with complex descriptive information and multi-step physical conversions, a substantial divergence in performance emerges. For metabolic rate fields requiring the conversion of historically mixed units (such as ($mL\;O_{2}\;g^{-1}\;h^{-1}$) into absolute energy power (W), the precision of the pure LLM baseline shows a severe degradation, dropping to 0.452 (95% CI: 0.417–0.487). Error analysis indicates that although LLMs possess a certain capability for numerical extraction (recall = 0.625, 95% CI: 0.594–0.656), they frequently produce floating-point calculation errors in subsequent processing and are highly prone to misinterpreting predicted intercepts from allometric regression models as empirically measured metabolic rates.

In contrast, because the proposed system strictly constrains the LLM to extract only raw numerical values and fully delegates computational authority to the Python sandbox for thermodynamic calculations and regression-equation circuit breaking, the F1 scores for measured body mass and normalized metabolic rate increase significantly to 0.928 (95% CI: 0.916–0.940) and 0.911 (95% CI: 0.895–0.927), respectively, demonstrating clear superiority over the baseline (*p* < 0.001). To further investigate the impact of data complexity and class imbalance as requested by the reviewers, we performed a stratified error analysis on the validation subset (Table 5). We categorized core fields into three complexity levels based on their syntactic structure, contextual dependency, and computational transformation requirements. The manually annotated instance counts reveal a natural imbalance across the 100-paper subset, in which taxonomic entities (*n* = 185) were more frequent than specialized physiological traits such as normalized metabolic rate (*n* = 76).

The results indicate a scientifically intuitive precision–recall trade-off: while False Positive (FP) rates remained consistently low across all levels, the False Negative (FN) rate reached 13.8% for the high-complexity field (normalized metabolic rate). Manual review suggests that the remaining false positives in this framework were mainly concentrated in extremely ambiguous scanning artifacts and misrecognition of handwritten annotations in early literature. These residual cases are associated with low-quality source material and irregular formatting, rather than failures of the core cross-page association, unit normalization, and deterministic conversion pipeline. Conversely, the elevation in false negatives arises from the system’s highly conservative “sandbox circuit breaker mechanism”. To minimize contamination of the final dataset, this deliberate design choice prioritizes data purity by automatically discarding ambiguous or heterogeneously formatted entries, resulting in the recall of the experimental group being slightly lower than its precision.

Table 6 further confirms that extraction performance decreases gradually with increasing field complexity. For low-complexity fields, represented by taxonomic entities, the framework achieves near-ceiling performance, with precision, recall, and F1 scores of 0.978, 0.965, and 0.971, respectively. For medium-complexity fields, including body mass and experimental temperature, precision remains high at 0.960, whereas recall decreases to 0.900, indicating a moderate recovery burden caused by contextual linkage and unit association. For high-complexity fields such as normalized metabolic rate, the system maintains strong precision (0.965) but recall decreases further to 0.862, reflecting the conservative filtering strategy of the sandbox mechanism when handling sparse variables requiring cross-page association and deterministic conversion. Overall, the macro-averaged performance remains strong, reaching 0.966 for precision, 0.907 for recall, and 0.935 for F1 score, demonstrating that the proposed framework sustains high extraction fidelity across heterogeneous complexity levels.

## 4. Discussion

Extracting functional traits from historical scientific literature has long been a major bottleneck in macroecology and comparative physiology. Despite the exponential growth of scientific publications, the vast majority of historical physiological data remain locked within unstructured PDF documents, complex multi-page tables, and supplementary images, preventing their systematic integration into modern large-scale ecological analyses. To unlock these extremely valuable historical records, this study develops and validates a neuro-symbolic bioinformatics framework. Unlike traditional natural language processing tools, which often suffer from context fragmentation and numerical hallucinations, our approach integrates visual layout perception with deterministic biostatistical reasoning. By decoupling text extraction from rigorous physical computation, we successfully achieve automated fine-grained mining of core physiological literature spanning the past 117 years. This pipeline constructs a high-precision, standardized physiological trait database covering 1632 chordate species, while also illustrating how principles that have been extensively discussed in related quantitative extraction fields, especially radiomics, can be adapted to historical bioinformatics literature mining to improve standardization, reproducibility, and downstream interpretability [24,25,26].

In terms of data quality control, the sandbox-level biostatistical filtering mechanism introduced by the system plays an important role. By strictly separating the non-deterministic generation of language models from the deterministic logic of the automated biostatistical pipeline, the system not only accurately standardizes heterogeneous physiological parameters, such as historically inconsistent units of oxygen consumption, into absolute thermodynamic power, but also effectively removes non-empirical, model-derived data through automated quality control and rigorous filtering mechanisms. This exclusion policy is intentionally limited to secondary quantities that are not direct empirical observations in the source papers. Although this conservative strategy improves measurement fidelity and protects the integrity of the final high-fidelity database, it may underrepresent scientifically meaningful derived information; future versions of the database will preserve such quantities in a separate metadata layer rather than discarding them outright. Although no temperature correction was applied, the main trend of allometric scaling remains significant due to the large sample size and broad taxonomic coverage. Rigorous manual sampling-based blind evaluation on the 100-paper validation subset shows that, compared to a pure large-model baseline, this framework achieves a macro-averaged F1 score of 0.935 across the core fields, with the strongest performance in low-complexity taxonomic entities and progressively lower recall in higher-complexity physiological traits. These results provide encouraging support for the reliability of the resulting high-fidelity database. From a cross-domain perspective, the present workflow is more analogous to radiomics-style quantitative pipelines than to conventional text mining alone. In radiomics, clinically or biologically useful quantitative features emerge only when heterogeneous raw inputs are processed through a reproducible pipeline comprising controlled preprocessing, standardized feature extraction, harmonization, and rigorous validation. Prior work in that field has shown that instability or ambiguity at any one stage can propagate downstream and compromise reproducibility. The same principle applies here. Our framework does not treat trait extraction from historical literature as a purely linguistic task; instead, it formalizes it as a quantitative measurement pipeline in which document perception, rule-based unit normalization, rejection of non-empirical quantities, and benchmark-based validation are tightly coupled. This framing helps situate our contribution within a broader class of established quantitative data extraction frameworks and clarifies why deterministic post-processing and provenance-preserving validation are central design choices rather than auxiliary technical details [24,25,26]. Compared with traditional document-extraction workflows that rely mainly on OCR and heuristic parsing, the present framework is better suited to degraded historical PDFs because it combines page-level visual perception with deterministic post-processing [8,20,21]. Likewise, compared with curated trait resources such as AnimalTraits v1.0.7 and PanTHERIA 1.0 (WR05 release, August 2008), the present database emphasizes record-level provenance, preserved experimental context, and source-traceable physiological observations rather than only consolidated species-level summaries [14,27].

Furthermore, macroecological consistency analyses, together with external benchmarking against PanTHERIA, support the biological plausibility and practical utility of the data generated by this framework. The extracted physiological parameters not only reproduce the classic allometric power-law relationships among basal metabolic rate, brain size, and body mass in chordates, but also preserve, with high fidelity, the extreme physiological limits within the long-tail distribution of natural traits. At the same time, benchmarking against PanTHERIA showed strong agreement on shared mammalian body-mass and metabolic-rate traits, while also highlighting an important difference in data structure: PanTHERIA provides species-level consolidated values, whereas our database preserves record-level provenance, original measurement context, and experimental metadata. Taken together, these findings suggest that the framework provides a practical route for improving the reliability of generative-AI-assisted scientific data extraction. Rather than replacing curated trait resources, it offers a complementary and scalable approach for constructing source-traceable physiological databases from historical literature, with potential utility for future comparative and evolutionary analyses. However, these allometric and extreme-value results should be interpreted as complementary plausibility checks rather than definitive proof of database superiority, because such broad biological relationships can remain robust even in the presence of moderate noise or sampling bias. In the present study, stronger support for data quality comes from the combined evidence of field-level extraction accuracy, deterministic standardization, retained record-level metadata, and concordance with PanTHERIA. Despite these encouraging results, this study still has certain limitations. First, to ensure absolute data purity, the automated filtering mechanism adopts an extremely conservative rejection strategy. This emphasis on precision inevitably sacrifices some recall, resulting in the exclusion of certain historically valuable but irregularly formatted data. Second, although the final output of 2192 valid records is constrained by the inherent scarcity of original in vivo physiological measurements in historical literature, it nevertheless serves as a robust proof of concept. In addition, although the 100-paper validation subset was designed to be stratified and representative, it remains small relative to the 117-year corpus. Future work should expand manual annotation and formalize reliability assessment on a larger sample. Moreover, although two annotators independently reviewed the validation subset and disagreements were resolved by consensus, a formal inter-annotator agreement statistic was not calculated in the current study. Reporting metrics such as Cohen’s kappa in future validation would further improve the transparency and reproducibility of the manual evaluation protocol. In addition, the allometric regressions were evaluated using OLS for descriptive plausibility checking rather than phylogenetically informed models such as PGLS. Future work should test these relationships using standard chordate phylogenies to assess the robustness of the scaling patterns under phylogenetic correction. Finally, although classical allometric relationships are useful for assessing biological plausibility, they are too robust to serve as the sole validation of database quality. Taken together, these limitations indicate that the present study should be understood as a reproducible proof of concept rather than a definitive endpoint for large-scale physiological database construction.

A further limitation is that the current implementation relies on proprietary GLM backends. Although the framework itself is modular and rule-based, future studies should evaluate open-source and alternative commercial models to further assess reproducibility and generalizability across backends. To address these limitations, we will optimize the extraction workflow to improve data recovery rates. We plan to introduce more efficient visual parsing models for preliminary literature screening to reduce computational overhead. In addition, we will explore more flexible validation mechanisms by incorporating semi-automated review strategies guided by domain experts, aiming to maintain high biological fidelity while further improving the recall of historical data. We will also retain scientifically meaningful derived variables in a dedicated metadata layer to reduce potential scope-related bias, thereby providing more comprehensive foundational data support for data-driven research in large-scale evolutionary modeling and global change ecology.

## 5. Conclusions

This study develops and evaluates a multimodal neuro-symbolic bioinformatics framework that integrates visual perception with rigorous biostatistical reasoning, providing an automated approach for unlocking unstructured historical records in macroecology and comparative physiology. By adapting the standard paradigm of decoupling semantic extraction and computation to the specific biophysical requirements of comparative physiology, the system significantly mitigates the numerical hallucinations typically associated with large language models, leveraging a deterministic logical sandbox for strict data quality control.

Across 117 years of historical literature, the framework automatically constructed a high-fidelity physiological trait database covering 1632 chordate species and achieved strong extraction performance (macro-averaged F1 = 0.935). External benchmarking against PanTHERIA further showed strong concordance on shared mammalian body-mass and metabolic-rate traits, while our database retained record-level provenance, original measurement context, and experimental metadata. Classical allometric relationships and extreme physiological values were also preserved in the extracted dataset, supporting its biological plausibility. Taken together, these results indicate that the proposed framework can generate a reproducible and complementary physiological database from historical literature rather than merely recover broad qualitative patterns.

Although the current system sacrifices some recall to ensure absolute data purity and faces computational overhead associated with high-resolution visual inference, its demonstrated potential for high-throughput data processing significantly lowers the barrier for laboratories to conduct large-scale physiological data mining. In the future, by introducing more efficient visual screening models and integrating domain-expert semi-automated review strategies, this framework is expected to play an important infrastructural role in data-driven research, facilitating large-scale cross-species comparative studies and evolutionary modeling under global climate change. With further optimization, this framework could be scaled to larger historical corpora and additional taxonomic groups, helping to close global trait-data gaps in biodiversity, macroecology, and climate-change research.

## Figures and Tables

**Table 1:** Species physiological trait records.

Data Category	Core Fields	Successfully Extracted Records	Coverage
Basic Taxonomic Information	Phylum, Class, Order, Family, Genus, Species	2192	100.0%
Core Physiological Traits	Body Mass	1968	89.8%
	Brain Volume	1672	76.3%
	Absolute Metabolic Rate	516	23.5%
	Mass-Specific Metabolic Rate	516	23.5%
Experimental Conditions and Parameters	Experimental Test Temperature	405	18.5%
	Sample Sex	436	19.9%
	Sample Size	2192	100.0%
Source Metadata	References and Publication Year	2192	100.0%
Total Processed Records	——	2192	——

**Table 2:** Comparison between the mammalian subset of our database and PanTHERIA.

Metric	Our Mammalian Subset	PanTHERIA	Interpretation
Taxonomic scope	Chordata-wide database; mammalian subset used here	Mammalia only	Our study-wide database is chordate-wide, whereas the benchmark resource is mammal-specific.
Data structure	Record-level	Species-level consolidated	Different granularity; our unit is a record, whereas the released PanTHERIA file reports one consolidated row per species.
Total mammalian records/rows	1020	5416	Observation-level rows in our database versus species-level rows in PanTHERIA WR05.
Total mammalian species	675	5416	Exact species-name counts after cleaning.
Shared mammalian species (exact binomial match)	541	541	80.1% of mammalian species in our database matched PanTHERIA by exact binomial name.
Unique mammalian species in our database	134	—	Conservative count; may include taxonomic or synonym mismatches because no synonym reconciliation was applied.
Body-mass records/species-level values available	935	3542	Our mammalian records with standardized body mass (kg) versus PanTHERIA species rows with non-missing adult body mass (g).
Shared species with directly comparable body mass	475	475	Species with our aggregated body mass and PanTHERIA adult body mass both available.
Metabolic-rate records/species-level values available	177	573	Our mammalian records with standardized absolute metabolic rate (W) versus PanTHERIA species rows with non-missing basal metabolic rate
Shared species with directly comparable BMR	81	81	Species with our aggregated metabolic rate and PanTHERIA BMR both available after unit harmonization to W.
Brain-size records available	839	—	Additional physiology dimension retained in our mammalian subset.
Experimental temperature retained	123	—	Metabolic observations in our mammalian subset with original experimental temperature retained; no corresponding field is present in the released PanTHERIA table.
Sample size retained	1020	—	All mammalian rows in our record-level database retain sample-size metadata, whereas no corresponding sample-size field is present in the released PanTHERIA table.
Literature-source linkage retained	Yes (full reference + publication year at record level)	Yes (species-level reference IDs in released table)	Both resources retain source linkage, but only our database retains it at record level with full citation text in the released file.
Original value and original unit retained	Yes	No (released table is standardized)	Our rows retain original body-mass and metabolic-rate fields and original unit strings; the released PanTHERIA table does not.

**Table 3:** Concordance between our mammalian subset and PanTHERIA on shared species.

Trait	Shared Species (*n*)	Pearson r (log10)	Spearman Rho (log10)	Median |Δlog10|	Median Fold Difference	OLS Slope (log10)	OLS Intercept (log10)
Body mass	475	0.988	0.987	0.081	1.21	0.972	−0.07
Metabolic rate (compared with PanTHERIA BMR)	81	0.996	0.995	0.01	1.02	1.009	−0.015

**Table 4:** Performance evaluation metrics table (Mean and 95% CI).

	Baseline (Pure LLM Without Sandbox)			Experimental Group (LLM + Python Sandbox)			Significance Testing
Evaluation Fields	Precision (P)	Recall (R)	F1 Score	Precision (P)	Recall (R)	F1 Score	*p*-Value (F1)
Taxonomic Entity	0.945 (0.930–0.960)	0.952 (0.933–0.971)	0.948 (0.934–0.962)	0.978 (0.968–0.988)	0.965 (0.953–0.977)	0.971 (0.962–0.980)	<0.05
Experimental Temperature	0.862 (0.840–0.884)	0.845 (0.820–0.870)	0.853 (0.832–0.874)	0.945 (0.930–0.960)	0.915 (0.896–0.934)	0.930 (0.916–0.944)	<0.01
Measured Body Mass	0.758 (0.731–0.785)	0.835 (0.813–0.857)	0.795 (0.776–0.814)	0.975 (0.964–0.986)	0.885 (0.868–0.902)	0.928 (0.916–0.940)	<0.001
Normalized Metabolic Rate	0.452 (0.417–0.487)	0.625 (0.594–0.656)	0.525 (0.498–0.552)	0.965 (0.951–0.979)	0.862 (0.840–0.884)	0.911 (0.895–0.927)	<0.001
Overall Average	0.754 (0.735–0.773)	0.814 (0.799–0.829)	0.780 (0.766–0.794)	0.966 (0.959–0.973)	0.907 (0.896–0.918)	0.935 (0.928–0.942)	<0.001

Tables Notes. Values are reported as mean performance across five independent validation samples (each N = 20), with 95% confidence intervals estimated using t-based intervals across the five sample-level values. Statistical significance was assessed using paired comparisons on the same sampled documents.

**Table 5:** Complexity stratification and error profile in the validation subset (*N* = 100 publications).

Core Field	Complexity Level	Manually Annotated Instances (*n*)	FP Rate (%)	FN Rate (%)
Taxonomic Entity	Low	185	2.2	3.5
Body Mass	Medium	142	2.5	11.5
Experimental Temperature	Medium	89	5.5	8.5
Normalized Metabolic Rate	High	76	3.5	13.8

Tables Notes. Complexity levels were defined a priori based on syntactic structure, contextual dependency, and computational transformation requirements. The validation subset was manually annotated by two independent researchers blinded to model outputs, and disagreements were resolved through consensus review. FP/FN rates were calculated from the 100-paper sample.

**Table 6:** Precision, Recall, and F1 Score stratified by field complexity.

Complexity Level	Precision (P)	Recall (R)	F1 Score
Low (Taxonomic Entities)	0.978 (0.970–0.986)	0.965 (0.955–0.975)	0.971 (0.964–0.978)
Medium (Body Mass & Temperature)	0.960 (0.948–0.972)	0.900 (0.883–0.917)	0.929 (0.918–0.940)
High (Metabolic Rate)	0.965 (0.952–0.978)	0.862 (0.840–0.884)	0.911 (0.898–0.924)
Overall (Macro-average)	0.966 (0.960–0.972)	0.907 (0.896–0.918)	0.935 (0.928–0.942)

Tables Notes. Performance metrics were aggregated by complexity level over the same manually annotated validation subset, and 95% confidence intervals were estimated using the same t-based procedure applied in Table 4.

## Data Availability

The detailed research protocol, data analysis plan, structured extraction template, and deterministic post-processing logic used in this study are available from the corresponding author upon reasonable request. The extracted benchmark tables and plotting source data used for comparative analyses can also be provided for verification and reproducibility purposes.
